# Utilization of magnesium-rich synthetic gypsum as magnesium fertilizer for oil palm grown on acidic soil

**DOI:** 10.1371/journal.pone.0234045

**Published:** 2020-06-16

**Authors:** Arolu Fatai Ayanda, Shamshuddin Jusop, Che Fauziah Ishak, Radziah Othman

**Affiliations:** Department of Land Management, Faculty of Agriculture, Universiti Putra Malaysia, Serdang, Selangor, Malaysia; Ohio State University South Centers, UNITED STATES

## Abstract

A study was conducted to determine the impact of applying different sources of Mg, namely kieserite, ground magnesium limestone (GML) and Mg-rich synthetic gypsum (MRSG) on an acid tropical soil, oil palm growth and production. Besides high amount of Mg and Ca, MRSG contains S. Exchangeable Ca in the untreated soil of the plantation was 0.64 cmol_c_ kg^-1^, but its critical level to sustain oil palm growth was 0.9 cmol_c_ kg^-1^. MRSG was applied in the plantation as Mg-fertilizer; however, since Ca is also a limiting nutrient, oil palm growth was correlated (r = 0.69) with Ca supplied by the MRSG. Mg needed to sustain oil palm production is normally supplied by kieserite. Its requirement can be met at a lower cost compared to that of the kieserite by using MRSG. Due to MRSG treatment, exchangeable Ca in the soil increased steadily to satisfy the requirement of oil palm for fruit bunches production. From the glasshouse and field study, it was observed that MRSG applied at 1.5 times the recommended rate gave results comparable to that of the kieserite. MRSG treatment resulted in the increase of soil pH to >5 that precipitated Al^3+^ as inert Al-hydroxides, which eventually enhanced oil palm seedlings growth. Thus, MRSG can also replace GML to increase soil pH and satisfy the Ca and Mg requirement of oil palm. It can be concluded that MRSG has the potential to be used as a source of Mg as well as Ca for oil palm grown on acidic soil.

## Introduction

In Malaysia and Indonesia, more than 5 and 10 million ha of their land area, respectively, are cropped to oil palm (*Elaeis guineensis*). The soils used for growing oil palm in these countries which are highly weathered Ultisols and Oxisols have insufficient nutrients for oil palm growth requirement. Hence, fertilizer application is crucial to sustaining yield production. One of the most important nutrients required by oil palm is Mg. It is a standard practice among oil palm plantations in the country to apply kieserite (MgSO_4_.H_2_O) as a source of Mg to sustain oil palm growth and production. Applying kieserite would also add S into the soil system; Mg is required for oil production in the fruitlets. Another equally important source of Mg giving comparable result to that of kieserite is dolomitic limestone [CaMg(CO_3_)_2_] [1], which is otherwise known as ground magnesium limestone (GML). GML releases Mg and Ca into the soil, which are needed to keep oil palm growing in the field without limitation.

Due to the high cost of fertilizer in oil palm production, it is imperative to look for a cheaper substitute to replace the expensive kieserite as a source of Mg. An industrial by-product called Mg-rich synthetic gypsum (MRSG), which is otherwise known as neutralization underflow (NUF) residue has been found to have beneficial properties which makes it a potential alternative to kieserite and GML as a source of Mg-fertilizer.

According to Golder Associates of Australia, this by-product contains magnesium hydroxide (17.1%), calcium hydroxide (4.3%) and calcium carbonate (2.3%), thus with a high pH of 8.8. MRSG is classified as Scheduled Waste (SW205) by the Department of Environment (DoE) which has given the permission for this material to be used in glasshouse study and field trial in an oil palm plantation. It is expected that the application of MRSG will improve the growth of oil palm, giving comparable result to that of the kieserite and GML and may as well go on to reduce soil acidity condition prevalent in the tropical region of the world where oil palm is mostly cultivated. This alongside the relatively low cost of MRSG may go on to prove its importance as an Mg source for use in oil palm plantation around the world. Thus, a study was conducted to determine the impact of applying different sources of Mg, which are Chinese kieserite, ground magnesium limestone and Mg-rich synthetic gypsum, on soil and oil palm growth/production.

## Materials and methods

### Characterization of MRSG

The MRSG used in the study was studied under Field Emission Scanning Electron Microscope (FESEM) and the elements present in it were identified using Energy Dispersive X-ray (EDX) attached to the FESEM. However, the mineralogical composition of the MRSG was determined by XRD analysis. To determine its elemental composition by ICP-OES, the MRSG was dissolved in a mixture of nitric acid and perchloric acid at the ratio of 3:1 with the addition of hydrogen peroxide [2], and elemental composition was determined by ICP-OES. A solubility experiment was also conducted to compare the solubility of Mg in MRSG, GML and kieserite. Alongside the MRSG sample, China kieserite and GML were respectively (based on the equivalent to 1 g of Mg) added in 300 mL of distilled water in duplicate. At each time point (5, 10, 20, 40 and 60 min), 20 mL of sample was drawn and analyzed with ICP. The Mg so determined was plotted against time.

The soil used for the nursery experiment was collected from the site of field experiment in Bera, Pahang, Malaysia (GPS 03.27362 N, 102.58044 E). The soil collected from the field was identified as Jempol Series using the criteria set by the Department of Agriculture Malaysia [3]. According to Soil Taxonomy [4], it belonged to the clayey, kaolinitic, isohyperthermic family of Typic Paleudults.

### Soil characterization

#### Soil physico-chemical analysis

Soil particle-size distribution determination was carried out using the pipette method. Soil pH was determined at the soil to water ratio of 1:2.5. Electrical conductivity (EC) was measured at soil to water ratio of 1:5. The CEC of the soil was determined by 1 M ammonium acetate buffered at pH 7. Basic exchangeable cations were extracted using 100 mL of 1 M ammonium acetate buffered at pH 7. The concentration of K, Ca, Mg and Na in the solutions was determined by Perkin Elmer Model AAS 3110 atomic absorption spectrophotometer (AAS). Total C in the soil was determined by dry combustion techniques, using LECO CR-412 Carbon Analyser. Available phosphorus was analyzed by the Bray and Kurtz II method [5]. Exchangeable Al was extracted using 1 M KCl and the Al in the extract was measured by Perkin Elmer Optima 8300, Norwalk, CT, USA, inductively coupled plasma-optical emission spectrometry (ICP-OES).

### Glasshouse study

#### Soil and soil sampling

**Fig 1:**
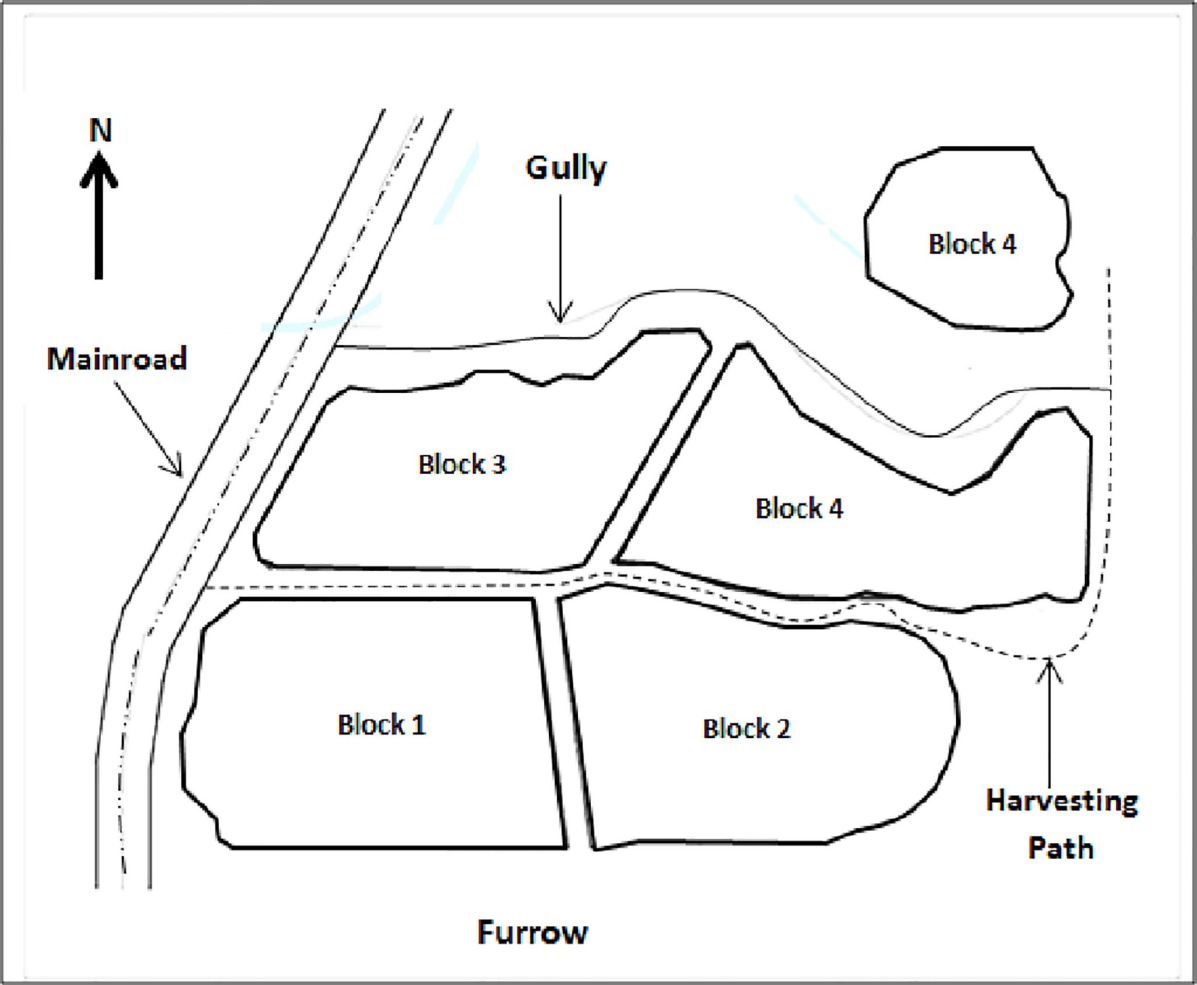
Diagrammatic presentation of the experimental blocks in the field.

The soil for the glasshouse study was taken from the site of field experiment in Bera, Pahang (Fig 1). The sample was taken from the topsoil (at 0–20 cm depth). The collected soil was immediately transported to Universiti Putra Malaysia (UPM) in Serdang to be processed for the use in laboratory analysis and glasshouse experiment.

#### Experimental design

The experiment was conducted at a glasshouse in the Faculty of Agriculture, UPM, Serdang for a period of 9 months. Three-month-old oil palm seedlings of the same size and/or height were planted in polybags having 20 kg soil; the experiment was conducted using Randomized Complete Block Design (RCBD), with 6 replications. The experimental units consisted of individual palm, with a total of 42 seedlings planted in the polybags. Based on the recommended magnesium requirement for oil palm nursery, an amount of 18 g of Mg was derived for each oil palm seedling grown on 20 kg soil and this was given as a one-time application alongside the NPK, for the duration of the experiment. The rate of NPK used for this study was recommended by the International Potash Institute [6]. The NPK fertilizers used for this experiment are 80.93g of Urea, 82.45 of g Triple superphosphate and 71.24g of Muriate of Potash. The treatment rates for the experiment are shown in Table 1. Kieserite and GML were included as treatments for the purpose of comparing their results with those of the MRSG.

**Table 1:** Label and details of the treatment used in the study.

Treatment	Description
T0	NPK without magnesium
T1	NPK+kieserite at the Mg recommended rate
T2	NPK+GML at the recommended rate of Mg
T3	NPK+MRSG at the recommended rate of Mg
T4	NPK+MRSG at 1.5 times the recommended rate of Mg
T5	NPK+ MRSG at two times the recommended rate of Mg
T6	NPK+MRSG equivalent to the amount of Ca in GML

T = Treatment, NPK = Nitrogen, Phosphorus, potassium (chemical fertilizer)

#### Sampling of soil and oil palm tissue

Sampling for soil and oil palm plant was conducted to determine the effect of treatment on soil chemical properties and the vegetative growth of the oil palm seedlings. This was carried out at 3, 6 and 9 months after planting. Based on standard procedure, oil palm tissues from frond number 3 were sampled for this purpose. The leaves were kept in an oven at 65°C overnight prior analysis. On the other hand, soil samples were air-dried and kept in containers while waiting for the chemical analyses to be carried out.

#### Measurement of growth parameters

Determination of growth parameters of the oil palm seedlings was carried out at 3 months interval. The parameters determined were plant height, the number of fronds, the weight of plant top, root weight and chlorophyll content.

#### Root growth

After harvesting, the roots were carefully separated from the aerial part of the oil palm seedling and washed with distilled water to remove soil particles. The roots were scanned to measure the total root length and surface area of the roots using the WinRHIZO-pro.

#### Analysis of oil palm tissue

Oil palm tissue analysis was carried out using the wet ashing method for the determination of C, N, P, K, Ca and Mg. Nitrogen and P in the solution were determined by AutoAnalyzer (QuickChem 8000 Series FIA System, Lachat Instruments, Loveland, USA), while K, Ca and Mg were determined by ICP-OES.

#### Soil analysis

The soil sampled at the 3-months interval was analyzed according to the methods described earlier in this paper (soil characterization).

### Field trial

#### Experimental design

The experimental area in the plantation was divided into 4 blocks (Fig 1), randomly arranged to satisfy the objectives of the field trial. In each block, the palms were marked accordingly to indicate the block, treatment and palm number.

The experimental design used was randomized complete block design (RCBD). There were five treatments applied as shown in Table 2. These treatments were replicated in four blocks (Fig 1), with each plot comprised six palms. Each treatment plot was separated by small ditches and the blocks were separated by larger drains to prevent trans-boundary movement of the applied materials. The NPK fertilizers required to sustain oil palm growth/production were applied at 6 months interval at the appropriate rates. MRSG, kieserite and GML applied four times in the experimental plots (for the two years duration of field trial) were broadcast and ploughed under to a depth of 15 cm within the weeded circle of each palm (1.5 m from the trunk base) at an interval of six months.

**Table 2:** The rate of GML, MRSG and kieserite applied in the research plots.

Treatment	Description	Rate of application
T0	Control 1: GML rate recommended by Felda Plantation	1.25 kg/palm application of GML
T1	(MRSG at equivalent amount of Mg in Kieserite)	1.1 kg/palm application of MRSG
T2	(MRSG at one-half the recommended rate of Mg in Kieserite)	1.45 kg/palm application of MRSG
T3	(MRSG at double the recommended rate of Ca in GML)	2.4 kg/palm application of MRSG
T4	(Control 2: Kieserite rate recommended by Felda Plantation)	0.5 kg/palm application of kieserite

#### Sampling of soil in the experimental plots

The sampling of soil in the experimental plots was carried out 3 times at 6 months interval. Soil samples for the analyses taken at 0–15 cm depth were within the weeded circle, using a soil auger.

#### Sampling and analysis of oil palm tissue

Frond 17 is the indicator tissue to show the nutrient status of matured palm [7]. Oil palm tissues from frond 17 were sampled twice at the interval of 6 months. Oil palm tissue analysis was carried out by wet ashing method to determine N, P, K, Ca, Mg, Al and Fe. N and P in the solution were measured by AutoAnalyser (QuickChem 8000 Series FIA System, Lachat Instruments, Loveland, USA), while ICP-OES was used to determine K, Ca, Mg, Al and Fe.

#### Harvesting of fresh fruit bunches (FFB) in the research plots

Harvesting of the FFB in the research plots was carried out every 2 weeks by the workers hired by the plantation owner under the supervision of the research team.

#### Determination of oil extraction rate

Oil extraction rate (OER) was determined by the method proposed by the National Research Centre of Oil Palm [8].

### Statistical analysis

Soil and oil palm tissue data obtained in the glasshouse study were statistically analysed, using SAS version 9.4. Analysis of variance was used to study the effects of the treatments on all the traits, while means comparison was done using the Least Significant Difference (LSD). Multiple regression analysis with stepwise selection method was conducted using SAS version 9.4. For the field trial, ANOVA for the analysis of variance for data on soil, oil palm tissue, OER and FFB yield was conducted using SAS version 9.4 (SAS Institute, Inc., Cary, N.C., USA), while means comparison was done using multiple T-test.

## Results and discussion

### Mineralogical composition of the MRSG

**Fig 2:**
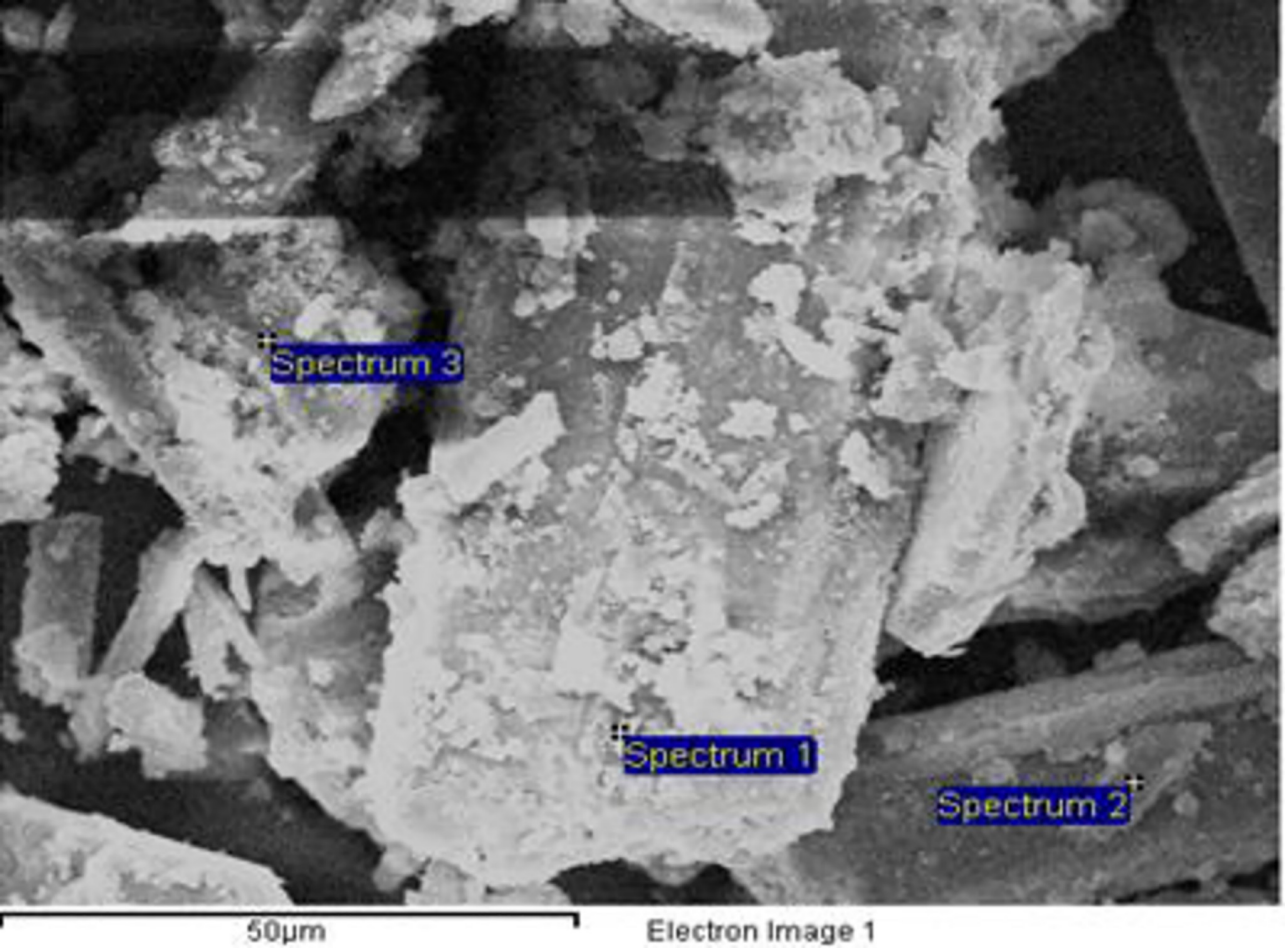
FESEM-EDX micrograph of the MRSG under investigation.

The result of the solubility test shows that the solubility of the tested materials is in order of: GML < MRSG < kieserite. The dominance of gypsum in the MRSG was evidenced by the presence of the acicular-shaped mineral observed under FESEM (Fig 2). The average chemical composition of the MRSG at any particular spot (e.g. at spectrum 1, 2 and 3) in the FESEM micrograph can be determined using EDX attached to the FESEM. The chemical contents of the MRSG determined by FESEM-EDX methodology (Fig 2) were the relative measurement, normalized to 100% and were not the absolute amount as determined by ICP-OES. In this study, spectrum analyses indicating the presence of certain elements were made. However, detailed elemental composition of the MRSG was tabulated based on ICP-OES analysis.

**Fig 3:**
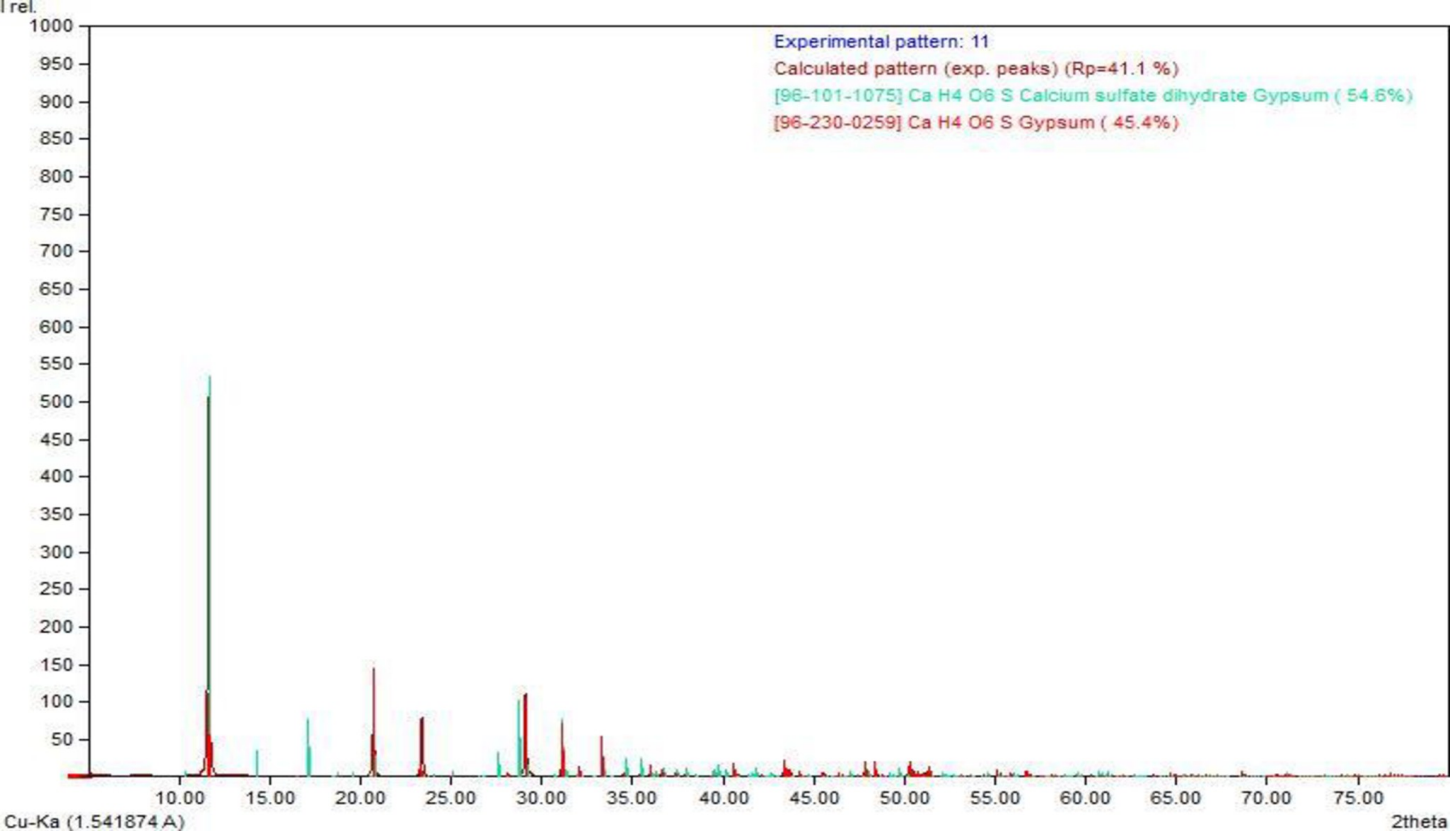
XRD diffractogram of the MRSG used in the study.

Mineralogical analysis of the MRSG (Fig 3) showed that it was mainly composed of gypsum (45.4%), proven by the d-spacing of 7.609 Å (2theta 11.63). There was also evidence for the presence of some calcite in the MRSG shown by the d-spacing of 3.036 Å (2theta of 29.41); however, the XRD peak was too weak to be clearly seen on the diffractogram (Fig 3). This finding is consistent with the detection of C in the MRSG by FESEM-EDX.

Plants nutrients and beneficial elements present in the MRSG are shown in Table 3. The most abundant macronutrient present was Ca (21%), followed by Mg (7%), with some P and K. The MRSG also contained some micronutrients (Mn and Zn), which are required in small amount to sustain oil palm growth. A small quantity but significant nonetheless was the presence of Se, which if sufficiently taken up by oil palm in the plantation and subsequently transported to its fruitlets, would have a far-reaching health benefit to human being consuming palm oil [9]. Si was detected by ICP-OES analysis, which was believed to exist in the form of amorphous silica. However, it cannot be confirmed by XRD analysis because it did not exist in the form of quartz, the crystalline form of SiO_2_. The finding that the silica in the MRSG existed as an amorphous material is consistent with that of Golder Associates of Australia. Silicon, in soil solution, if taken up by oil palm is able to prevent and/or cure certain disease prevailing in the plantations [10].

**Table 3:** Plant nutrients and beneficial elements present in the MRSG.

Group	Element	Amount (mg/kg)
Macronutrient	P	234
	Ca	209871
	Mg	71483
	K	49.39
Micronutrient	Fe	1368
	Mn	1175
	Zn	38.68
	Cu	127
Beneficial element	Si	19.14
	Se	0.35

### Glasshouse study

#### Baseline properties of the soil before treatment

The soil used for the glasshouse study was collected from the field experimental site. This soil was classified as a clayey, isohyperthermic family of Typic Paleudult [3] and named as soil of the Jempol Series according to the Malaysian system for soil classification. The physico-chemical properties of the soil are as shown in Table 4.

**Table 4:** The physico-chemical properties of the soil used in the experiment.

Soil properties		**Nutrient ranking for oil palm (High)
Sand (%)	7.34	
Silt (%)	14.88	
Clay (%)	75.78	
Textural class	Clayey	
pH (water)	4.92	4.2–5.5
Av. P (mg kg^-1^)	3.92	20–25
Total N (%)	0.13	0.15–0.25
Total C (%)	1.05	1.5–2.5
Exc. Ca (cmol_c_ kg-^1^)	0.64	
Exc. Mg (cmol_c_ kg-^1^)	0.23	0.25–0.30
Exc. K (cmol_c_ kg-^1^)	0.13	0.25–0.30
Exc. Na (cmol_c_ kg-^1^)	0.04	
Exc. Al (cmol_c_ kg-^1^)	1.41	
CEC	7.11	15–18
Cu (mg kg^-1^)	64.50	
Fe (mg kg^-1^)	7152.50	
Mn (mg kg^-1^)	283.50	
Zn (mg kg^-1^)	67.70	

Av. P = available phosphorus, N = Nitrogen, C = Carbon, CEC = Cation Exchange Capacity, Exc. K = exchangeable potassium, Exc. Ca = exchangeable calcium, Exc. Mg = exchangeable magnesium, Exc. Na = exchangeable sodium, Exc. Al = exchangeable aluminum.

** Source: Goh et al. (2003).

The soil used in the experiment is acidic with a pH of <5 (Table 4); however, according to *Goh et al*. [11] it is still within the suitable soil pH range for oil palm cultivation. The exchangeable magnesium in the soil was 0.23 cmol_c_ kg^-1^. This concentration is considered as moderate for oil palm growth. The CEC of the Ultisol under study was within the level expected for a typical highly weathered soil in the tropics (Table 4). The available phosphorus in the soil was very low (Table 4). Low P availability could be due to its immobilization via specific adsorption by Fe and Al oxides, forming insoluble Fe-P and/or Al-P compound [12]; note that Fe was very high in the soil. The pKa of Al is 5; hence, soil solution pH will go towards 5 to achieve the state of equilibrium. High organic matter content as reflected by the high nitrogen and carbon in the topsoil was thought to be due to the contribution from the felled fronds and empty fruit bunches that were previously laid down in the inter-rows of the oil palm.

#### Effects of MRSG treatment on soil

Exchangeable Mg in the soil of the glasshouse study at harvest (month 9) indicated that the level of Mg in soil treated with MRSG is comparable to that of kieserite application (Table 9). Exchangeable Mg and Ca in the topsoil of the plantation was 0.23 and 0.64 cmol_c_/kg soil, respectively (Table 5), and these values were below the sufficiency level for the optimal oil palm growth [13]. Mg or even Ca required by oil palm can be supplied by GML application; however, the standard practice of supplying Mg for oil palm consumption is by applying kieserite. Calcium is mostly neglected in oil palm nutrition since its deficiency has been rarely reported [14]. However, in the past, Ca was added to the soil through liming. The large hectare of land cultivated to oil palm has made liming a very expensive practice. Despite that oil palm is an acid-tolerant plant species [15], which can tolerate soil acidity below pH of 5. This study showed that raising soil pH to higher level enhanced the growth of oil palm. Normally, this is only achieved through liming which has been found to be expensive for oil palm plantations. The use of MRSG as a magnesium fertilizer adds a valuable amount of Ca into the soil. This Ca also raise soil pH which is good for the growth of oil palm seedlings. Thus, there is a justification to raise the pH of the Ultisols in Malaysia or Indonesia for oil palm cultivation to a higher level where it is expected that oil palm will perform even better.

**Table 5:** Soil pH and exchangeable Ca and Mg as affected by MRSG treatments.

Trt	Month 3			Month 6			Month 9		
	pH	Exch Ca	Exch Mg	pH	Exch Ca	Exch Mg	pH	Exch Ca	Exch Mg
	cmol_c_/kg								
T0	5.32^f^	0.72^d^	0.28^e^	5.27^e^	0.49^f^	0.28^e^	5.23^e^	0.49^d^	0.28^e^
T1	6.02^e^	0.86^cd^	0.39^d^	6.19^d^	0.76^c^	0.45^c^	6.17^d^	0.82^c^	0.51^b^
T2	6.48^b^	1.03^c^	0.31^e^	6.41^b^	1.25^b^	0.35^d^	6.25^b^	1.28^a^	0.36^cd^
T3	6.18^d^	1.36^b^	0.34^ed^	6.20^d^	1.06^cd^	0.34^d^	6.18^d^	0.92^c^	0.34^d^
T4	6.20^cd^	1.39^ab^	0.52^c^	6.29^c^	1.02^d^	0.47^bc^	6.28^cd^	1.16^b^	0.42^c^
T5	6.76^a^	1.61^a^	0.71^a^	6.83^a^	1.44^a^	0.61^a^	6.99^a^	1.39^a^	0.61^a^
T6	6.22^c^	0.47^ab^	0.63^b^	6.34^c^	1.22^bc^	0.50^bc^	6.36^bc^	1.32^a^	0.47^b^

Means followed by different letter within same column are significantly different at p≤0.05

Application of MRSG on the soil resulted in a significant increase in its pH, exchangeable Ca and exchangeable Mg (Table 5). Soil pH of the control treatment was already close to 5, which is rather unusual for the Ultisol in Peninsular Malaysia [13]. There could be possible contamination of the control plots by running water (run-off) from the treated plots. Nevertheless, the results showed that soil pH increased further with increasing rate of the MRSG treatment having values exceeding 6 or higher, with a concomitant increase in exchangeable Ca and Mg. This means that applying MRSG improve soil fertility significantly which is believed would enhance the growth of the oil palm seedlings planted under glasshouse conditions.

#### Effects of MRSG treatment on oil palm seedling

The growth of oil palm seedlings in the glasshouse as seen from height, stem diameter, root length and root surface area was significantly enhanced by the addition of MRSG, which gives result comparable to other source of Mg-fertilizer (Table 6). In terms of the vegetative growth of the oil palm seedlings, MRSG treatments gave comparable results to that of the kieserite. This is encouraging as it indicates the possibility of using MRSG as a replacement of kieserite for Mg source (Mg-fertilizer) to sustain oil palm seedling growth.

**Table 6:** Effects of treatments on height, stem and root diameter on oil palm seedling.

Treatment	Height (cm)	Stem diameter (mm)	Root length cm/plant	Root Surface area cm^2^/palm
T0	131.05b	75.00d	18684b	3296.6c
T1	164.66a	82.10abc	21856a	3371.2bc
T2	161.57a	87.35a	23120a	3487.1bc
T3	166.01a	80.61bcd	22429a	3353.8c
T4	164.00a	84.40ab	21543a	3746.9ab
T5	172.83a	77.33cd	21924a	3936.4a
T6	163.83a	84.08ab	23615a	3650.9abc

Means followed by different letter within same column are significantly different at p≤0.05

The Mg content in frond 3 of the oil palm seedlings was within the sufficiency range for their healthy growth (Table 7). In the case of Ca content, the values were higher than the sufficiency level for oil palm requirement, based on the standard requirement proposed by *Von Uexkull and Fairhurst* [16] and *Fairhurst and Hardter* [17]. This indicates that for both Mg and Ca, the higher their contents in the soil due to application of MRSG, the higher the uptake was by the oil palm seedlings.

**Table 7:** Mg and Ca content in frond 3 of the oil palm seedlings.

Nutrient	Data from this study	Nutrient sufficiency level for oil palm*
	(%)	
Magnesium	0.29–0.45	0.30–0.42
Calcium	0.81–1.21	0.50–0.70

*Von Uexkull and Fairhurst (1991) and Fairhurst (2003)

The height of oil palm seedlings growing in the glasshouse was plotted against exchangeable Mg where it was observed that there was no significant correlation between the height of oil palm and the level of exchangeable magnesium in the soil. However, when the plant height was plotted against the level of exchangeable Ca, a significant correlation was obtained. This suggests that calcium was the more limiting nutrient in the soil.

### Growth calibration curve and correlation study

**Fig 4:**
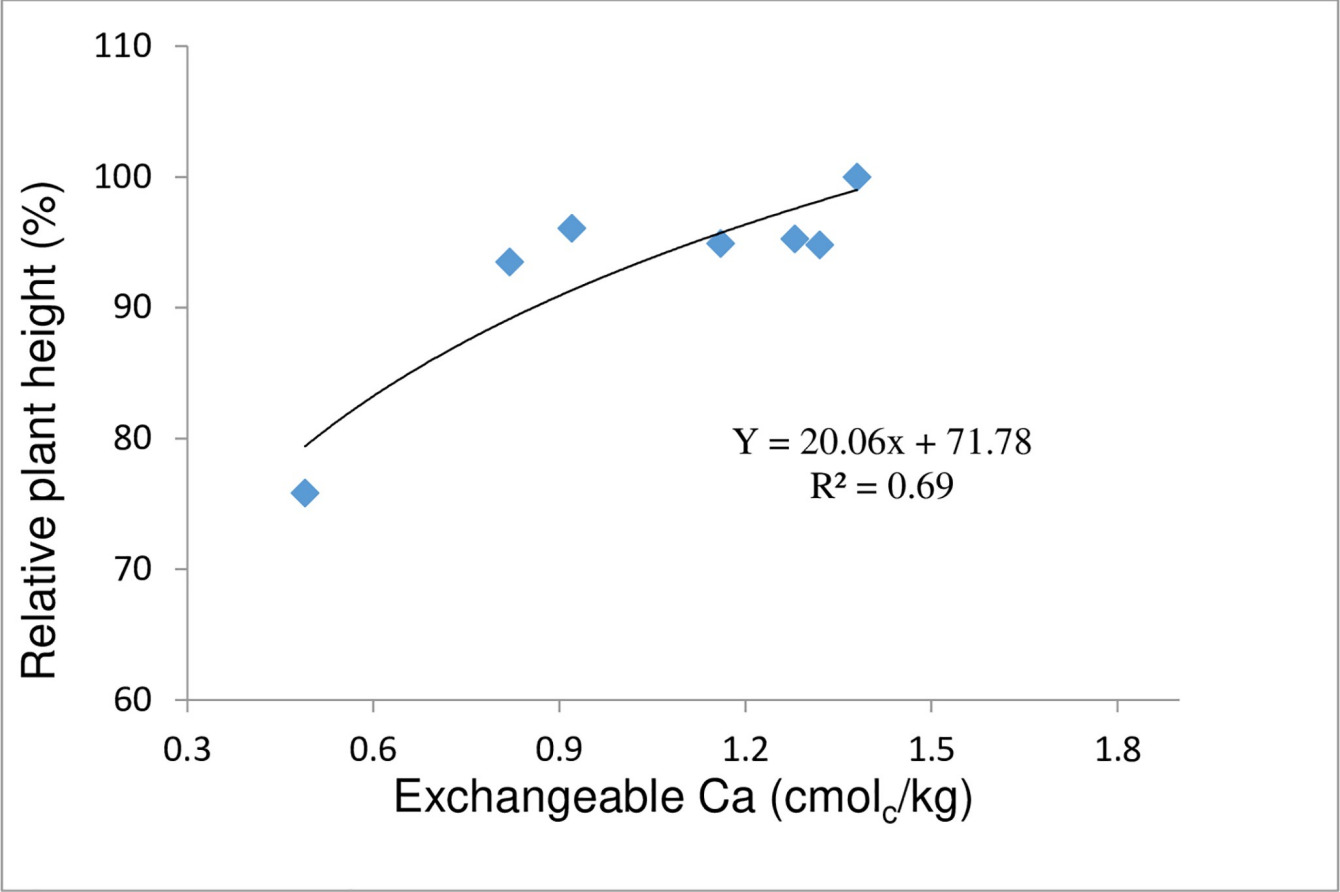
Relationship between relative plant height and exchangeable Ca.

The relative plant height was used as an indicator of plant growth. The relative plant height (%) values were then calculated and subsequently plotted against exchangeable Ca in order to determine the critical level of exchangeable Ca to sustain oil palm growth (Fig 4). It was found that the critical exchangeable Ca value estimated in this way was 0.9 cmol_c_/kg (value taken at 90% relative plant height). This means that the topsoil exchangeable Ca of 0.64 cmol_c_/kg in the plantation was insufficient for the oil palm growth.

Exchangeable Ca of more than 0.9 cmol_c_/kg is rather uncommon for the Ultisols in Peninsular Malaysia under continuous oil palm cultivation. As such, exchangeable Ca level of the soil in the plantation has to be raised accordingly via agronomic means. This study showed that at this level of exchangeable calcium, soil pH increases to level above 5.

Multiple regression analysis of the soil pH, exchangeable calcium and exchangeable magnesium showed that a significant relationship existed between these variables and the height of oil palm seedlings, with R^2^ equal to 0.9215. This means that about 92% of the relationship can be explained by the regression line. Using this statistical method, it was confirmed that soil pH was the most factor controlling the growth of oil palm seedlings based on its height. Further, the result showed that soil pH was positively correlated with the exchangeable Ca, which means that as Ca in the soil increased, soil pH was raised to a level dependent on the rate of MRSG applied. Thus, as shown by the plant height, it can be assumed that if soil pH, exchangeable Ca and exchangeable Mg are sufficiently increased, the growth of the oil palm seedlings will be enhanced significantly. This can be done via the application of MRSG at the appropriate rate and time.

It is known that Al^3+^ in soil solution will be precipitated as inert Al-hydroxides at the pH above 5. For the topsoil of Jempol Series under investigation, even though the field soil pH was slightly below 5, the exchangeable Al was still high (1.41cmol_c_/kg). The result of the glasshouse study showed that soil pH was significantly correlated with the height of oil palm seedlings.

It follows that the increase in soil pH had resulted in better growth of the oil palm seedlings. Soil pH increase after MRSG application was partly due to the addition of hydroxyl ions released by Mg and Ca hydroxides contained in it. It was also promoted slightly by the reaction of calcite, a liming agent, present in the MRSG.

### Field trial

#### Baseline properties of the studied area

As mentioned earlier, the experimental area in the oil plantation was divided into four blocks (Fig 1), where trees in each block were labelled accordingly to indicate the block, treatment and palm number. Results of the soil survey in the study area shows that only Jempol series was identified, evenly distributed throughout the 4-ha oil palm plantation. The baseline physico-chemical properties are shown in Table 8.

**Table 8:** The baseline physico-chemical properties of the soil in the field experiment area.

Soil parameter	Block				
	1	2	3	4	S.D
Sand (%)	11.00	9.00	7.00	5.00	2.39
Very Coarse sand 2–1 mm	1.16	1.12	0.50	0.22	0.43
Coarse sand 1.0–0.5 mm	1.46	1.40	1.42	0.72	0.33
Medium sand 0.5–0.25 mm	2.28	1.89	1.32	1.15	0.48
Fine sand 0.25–0.125 mm	2.98	2.20	1.96	1.52	0.57
Very fine sand 0.125–0.063 mm	3.12	2.39	1.80	1.39	0.70
silt (%) 0.063–0.002 mm	14.00	17.00	17.00	10.00	3.07
Clay (%) <0.002 mm	75.00	74.00	76.00	86.00	5.15
Organic matter (%)	6.63	8.72	7.34	7.27	0.81
Organic carbon (%)	1.74	2.10	1.86	1.77	0.15
pH Water (1:2.5)	4.33	4.54	4.44	4.40	0.08
Electrical Conductivity (μS/cm)	2480	2460	2390	2410	38.91
Exchangeable Al (cmol_c_/kg)	0.90	0.05	0.06	0.10	0.38
Exchangeable H (cmol_c_/kg)	0.70	0.40	0.40	0.40	0.14
Exchangeable Ca (cmol_c_/kg)	0.97	1.20	1.24	0.87	0.17
Exchangeable Mg (cmol_c_/kg)	0.98	1.12	0.80	0.71	0.17
Exchangeable Na (cmol_c_/kg)	0.06	0.05	0.04	0.05	0.01
Exchangeable K (cmol_c_/kg)	0.61	0.26	0.24	0.50	0.17
Cation exchange capacity (cmol_c_/kg)	4.22	3.04	2.73	3.04	0.61
Total N (%)	2.36	2.30	2.14	2.30	0.09
Total S (%)	0.16	0.11	0.15	0.10	0.03
Total C (%)	2.17	3.28	2.79	2.54	0.43

*S.D = standard deviation

#### Effects of MRSG application on soil chemical properties

Soil data in March 2016 showed that there was no significant difference in the exchangeable Mg or Ca between treatments over the period of the study (Table 9). Application of MRSG produced results comparable to that of the kieserite in terms of supplying Mg. Note that MRSG application also supplied Ca, which kieserite did not. The amount of Mg and Ca in the soil was sufficient for the healthy growth of the oil palm in the plantation.

**Table 9:** Effects of treatments on soil pH, exchangeable Mg, exchangeable Ca and CEC.

TRT	At 6-month				At 12-month				At 18-month			
	pH	Mg	Ca	CEC	pH	Mg	Ca	CEC	pH	Mg	Ca	CEC
		cmol_c_ kg-^1^				cmol_c_ kg-^1^				cmol_c_ kg-^1^		
T0	-	0.46a	1.05a	9.59ab	4.27a	0.87a	1.70a	15.77a	4.37a	1.03a	0.27a	10.67a
T1	-	0.46a	1.03a	10.19ab	3.99a	0.25a	1.20a	15.79a	4.26a	0.64a	0.24a	11.17a
T2	-	0.38a	1.15a	11.54a	4.39a	0.36a	1.13a	15.77a	4.10a	0.71a	0.24a	11.21a
T3	-	0.60a	1.52a	10.54ab	3.90a	0.32a	1.07a	16.55a	4.40a	0.88a	0.26a	10.71a
T4	-	0.42a	0.98a	14.43a	4.31a	0.59a	1.25a	17.84a	4.26a	0.78a	0.24a	10.96a

Means followed by different letter within the same column are significantly different at p≤0.05

The respective soil pH at 12 and 18 month (Table 9) was 3.9–4.3 and 4.1–4.4, without any difference among treatments. There seems to indicate that there was a slight increase in soil pH with time, which can be regarded as an ameliorative impact of applying MRSG on the soil occurring due to addition of Ca from this material. Soil pH increase resulting from MRSG application was partly due to the addition of hydroxyl ions released by Mg and Ca hydroxides contained in it. It was also promoted slightly by the reaction of calcite, a liming agent, present in the MRSG. It is possible that continuous application of this material, in the long run, would reduce soil acidity even further. The critical soil pH for oil palm in Malaysia is 4.3 [15]; so in terms of soil pH, oil palm in the plantation was able to sustain its growth. It has been known that increase in soil pH to a level above 5 would lower soil exchangeable Al upon which other nutrients will become available in the soil for plant uptake [18].

The CEC of the soil was within the range expected for a typical highly weathered Ultisol containing sufficient amount of organic matter. After 18 months of experimental duration (Table 9), total soil C was 1.59–2.08%. Organic C in the soil was high, which was due to the proper soil/agronomic management of the plantation. Oil palm plantations in the country place cut fronds and empty fruit bunches in the inter-rows of the palms. When these materials are decomposed and/or mineralized, C and plant nutrients are returned to the soil which eventually enhances soil fertility. The CEC of the soil at 6 month of experimental duration was 9.59–14.43, while at 18 month, it was 10.67–11.21 cmol_c_/kg (Table 9). Based on the afore-mentioned chemical attributes, the soil was in good condition for oil palm growth and/or production.

#### Effects of MRSG application on oil palm

Effects of MRSG application on nutrient content in frond 17, fresh fruit bunches and fruitlet yields are discussed below.

*Effects of MRSG application on nutrient content*. A significant difference was observed for Ca and Mg in frond 17 among treatments (Table 10). The Ca and Mg level were within the sufficient range (NSR) for matured oil palm under production, based on the requirement levels proposed by *Von Uexkull and Fairhurst* [16] and *Fairhurst and Hardter* [17]. The concentration of NPK by oil palm was not affected by MRSG treatments. It means that MRSG application has similar effect to that of the kieserite or GML (control treatments), in terms of nutrient uptake by the oil palm. This finding proves that MRSG is as good as kieserite in terms of supplying Mg for the requirement of oil palm although it may take longer time to release the nutrient into the soil unlike in kieserite. MRSG is thus a good Mg-fertilizer as well as a source of Ca which helps sustain oil palm growth and/or production.

**Table 10:** Chemical composition of oil palm frond at 6-month of experimental duration.

Treatment	C	N	P	K	Ca	Mg	Al	Fe
	%						mg/kg	
T0	44.56a	2.32a	0.07a	1.10a	0.59ab	0.25a	77.73a	96.71a
T1	44.81a	2.37a	0.07a	1.00a	0.57ab	0.20a	126.66a	99.24a
T2	44.44a	2.32a	0.07a	0.85a	0.73a	0.25a	102.65a	99.49a
T3	44.68a	2.44a	0.07a	0.91a	0.77a	0.27a	65.10a	87.09 a
T4	44.43a	2.44a	0.07a	1.08a	0.63a	0.23a	103.18a	106.6 a
NSR*	n.a	2.24–2.97	0.08–0.14	0.78–0.91	0.74–1.53	0.25–0.98		

Means followed by different letter within the same column are significantly different at p≤0.05.

NSR*: Nutrient sufficiency range for matured oil palm

The Ca/Mg ratio in oil palm frond 17 is a matter of concern among plantation owners in Malaysia. The ratio in the leaves should be within 1.5–3.0 range [17]. Therefore, higher Ca is needed compared to that of Mg to sustain oil palm growth/production. The respective Ca/Mg ratio for T0, T1, T2, T3 and T4 in the current study were 2.4, 2.9, 2.9, 2.9 and 2.7. Hence, there was no Ca-Mg imbalance due to MRSG treatment. Treating the soil with MRSG or kieserite resulted in similar uptake of Ca and Mg by the oil palm.

The chemical composition of the oil palm tissue at 6 months after the second MRSG application is presented in Table 11. Again there were no significant differences among treatments for all the parameters measured. However, Ca and Mg in the tissues were a bit lower than the sufficient range for normal oil palm growth.

**Table 11:** Chemical composition of the oil palm frond at 6 month after second MRSG application.

Treatment	C	N	P	K	Ca	Mg	Al	Fe
	%						mg/kg	
T0	45.12 a	1.80 a	0.13 a	0.61 a	0.41 a	0.18 a	188.63 a	188.50 a
T1	45.38 a	1.96 a	0.12 a	0.56 a	0.49 a	0.16 a	141.75 a	146.88 a
T2	45.17 a	2.11 a	0.11 a	0.47 a	0.51 a	0.20 a	231.00 a	234.13 a
T3	45.42 a	1.87 a	0.12 a	0.46 a	0.41 a	0.18 a	275.38 a	271.00 a
T4	45.90 a	1.88 a	0.12 a	0.56 a	0.36 a	0.17 a	180.63 a	201.75 a
NSR*	n.a	2.24–2.97	0.08–0.14	0.78–0.91	0.74–1.53	0.25–0.98		

*Effects of MRSG application on FFB and fruitlets yield*. Table 12 gives the mean values for the number and weight of FFB by month from June to November 2016. The study showed that there was no significant difference in the number and weight of FFB among treatments. However, the means separation by month showed significant yield increment towards the end of 2016. This was mainly due to the effect of increased rainfall occurring towards the end of the year. This finding proves that oil palm growing in the field requires adequate amount of water for its healthy growth and the production of fruit bunches [19].

**Table 12:** FFB yield at 9–14 months of experimental duration.

Month	No. of FFB					Weight of FFB (kg)				
	T0	T1	T2	T3	T4	T0	T1	T2	T3	T4
9	5 aB	7 aB	5 aB	5 aB	9 aB	112.85 aBC	145.53 aBC	95.50 aBC	107.45 aBC	180.60 aBC
10	7 aB	4 aB	4 aB	4 aB	5 aB	153.48 aC	102.63 aC	65.40 aC	75.13 aC	106.35 aC
11	6 aB	7 aB	5 aB	5 aB	6 aB	138.05 aBC	149.20 aBC	95.75 aBC	109.25 aBC	130.48 aBC
12	5 aB	7 aB	4 aB	5 aB	4 aB	103.10 aC	155.63 aC	95.87 aC	102.28 aC	94.90 aC
13	10 aA	10 aA	8 aA	9 aA	10 aA	191.98 aAB	191.83 aAB	142.35 aAB	155.25 aAB	202.55 aAB
14	8 aB	11 aB	9 aB	10 aB	7 aB	180.10 aA	232.60 aA	172.95 aA	218.03 aA	166.23 aA

Means followed by different small letter within the same row are significantly different at p≤0.05 (between treatments)

Means followed by different capital letter within the same column are significantly different at p≤0.05 (between months)

The FFB harvested from January to August 2017 showed slight fluctuation between months (Table 13). However, there were no significant differences in FFB yield between treatments for each month. There seems to be an indication that T3 (MRSG treatment) produced higher FFB weight compared that of the control treatments (T0 and T4) in January. It is possible that MRSG application could enhance the growth of the oil palm that eventually increased the FFB weight. This phenomenon could have been due to the enhancement of soil fertility shown by the increase in soil pH as well as Mg and/or Ca or it could even be due the presence of extra micronutrients or essential elements (Table 7).

**Table 13:** FFB yield data for 16-23months of experimental durations.

	No. of FFB					Weight of FFB (kg)				
Month	T0	T1	T2	T3	T4	T0	T1	T2	T3	T4
16	6 abAB	9 aA	9 aAB	9 aA	5 bAB	119.00 bcAB	180.90 abcA	205.94 abA	222.13 aA	95.05 cAB
17	4 aB	5 aB	5 aB	5 aB	4 aB	92.23 aAB	99.85 aB	120.40 aB	94.88 aB	77.88 aC
18	10 aA	8 aA	10 aA	9 aA	8 aA	228.88 aA	183.80 aA	222.45 aA	197.30 aA	174.18 aA
19	9 aAB	7 a AB	7 aB	9 aA	6 aAB	199.60 aA	139.73 aB	143.50 aAB	173.60 aAB	127.33 aAB
20	13 aA	9 abA	12 aA	6 bA	7 bA	257.68 aA	158.65 abAB	251.15 aA	130.28 bAB	140.60 abA
21	5 aB	6 aB	5 aB	6 aA	3 aB	111.85 aAB	124.45 aB	94.98 aB	116.78 aB	78.05 aC
22	4 aB	5 aA	2 aC	3 aB	4 aB	96.55 abAB	128.25 aB	36.80 bC	66.55 abC	107.98 abAB
23	4 aB	5 aB	5 aB	3 aB	5 aAB	75.05 aB	96.08 aB	112.08 aB	64.40 aC	105.10 aAB

Means followed by different small letter within the same row are significantly different at p≤0.05 (between treatments). Means followed by different capital letter within the same column are significantly different at p≤0.05 (between months)

Table 14 shows FFB yield harvested at 18 months of experimental duration. There was no significant difference in FFB weight and the number of fruitlets between treatments with MRSG and control treatments. Thus, treating the Ultisol in the plantation with MRSG gives comparable results to that of kieserite, in terms of weight of the oil palm fresh fruit bunches and the number of fruitlets in each fruit bunch.

**Table 14:** Weight of one fresh fruit bunch, the number of fruitlets per fruit bunch and the weight of each fruitlet at 18 month of experimental duration.

Treatment	FFB weight (kg)	Fruitlet weight (g)	Number of fruitlets
T0	20.98 a	8.94 a	1831 a
T1	22.56 a	8.45 a	2197 a
T2	22.06 a	8.88 a	1928 a
T3	20.42 a	10.82 a	1662 a
T4	22.03 a	8.33 a	1958 a

Means followed by different letter within the same column are significantly different at p≤0.05

***Effect of MRSG application on the oil extraction rate***. Fruitlets for the OER analysis were sampled about 2 years after the first MRSG application on the soil to make sure that the interpretation of the results obtained was valid. The analysis of the OER showed that there was no significant difference among treatments, with values ranging from 16.3 to 22% (Table 15). The OER values due to MRSG treatments were comparable to those obtained by the commercial plantations in Malaysia. It seems that treating the soil with MRSG produced higher OER compared to that of the kieserite or GML.

**Table 15:** Oil extraction rate as affected by treatments.

Treatment	Material	Rate (kg/palm)	OER (%)
T0	GML	1.25	16.3 a
T1	MRSG	1.10	17.1 a
T2	MRSG	1.45	22.0 a
T3	MRSG	2.40	18.6 a
T4	Kieserite	0.50	17.2 a

Means followed by different letter within the same column are significantly different at p≤0.05

Being a by-product of a chemical plant producing rare earth, MRSG is cheaper compared to that of the GML. MRSG is available in large quantity as long as the chemical plant in Malaysia is in operation. The environmental impact of MRSG application in this trial has been published by *Sahibin* et al. [20].

The contribution of kieserite, ground magnesium limestone and Mg-rich synthetic gypsum tested in the study towards the enhancement of soil fertility is summarized in Table 16. It is clear that MRSG is superior in terms of macro- and micronutrient supply for the requirement of oil palm compared to those provided by kieserite or GML. According to *Shamshuddin and Ismail* [21], GML produced in Malaysia contained some Mn and Zn. Kieserite does not change soil pH, but both GML and MRSG do. However, MRSG contains S (which is required for oil production in the fruitlets) and it is cheaper than kieserite or GML. Thus, in general, MRSG is considered to be comparable or better than kieserite.

**Table 16:** The key differences in the chemical properties among the tested fertilizers.

Fertilizer	Formula	Macronutrient	Micro/Essential nutrient	Change in pH
Kieserite	MgSO_4_.H_2_O	Mg, S	-	No change in pH
GML	Ca,Mg(CO_3_)_2_	Ca, Mg	Mn, Zn	Increase soil pH
MRSG	CaSO_4_.2H_2_O + Mg	Ca, Mg, S	Mn, Zn, Se	Increase soil pH

## Conclusions

The study showed that the growth of the oil palm was enhanced by the increase of Ca in the soil, resulting from MRSG application. The critical exchangeable Ca for the healthy growth of the oil palm was 0.9 cmol_c_/kg, which was higher than that present in the Ultisol under investigation. The possible problem of Ca deficiency in the soil can be overcome effectively by the continuous MRSG application for a long time. Normally, Mg required to sustain oil palm growth and/or production is supplied by applying kieserite. The current study showed that it is better or cheaper to supply Mg via MRSG application compared to that of the kieserite. MRSG not only is a reliable Mg and Ca source, but also exhibits liming properties and thus is an effective soil ameliorant. In the long run, its application would enhance soil fertility and eventually sustain oil palm production on the Ultisol either in Peninsular Malaysia or anywhere else in tropical regions.
